# Contribution of Atmospheric Rivers to Antarctic Precipitation

**DOI:** 10.1029/2022GL100585

**Published:** 2022-09-14

**Authors:** Michelle L. Maclennan, Jan T. M. Lenaerts, Christine Shields, Jonathan D. Wille

**Affiliations:** ^1^ Department of Atmospheric and Oceanic Sciences University of Colorado Boulder Boulder CO USA; ^2^ National Center for Atmospheric Research Boulder CO USA; ^3^ Institut des Géosciences de l’Environnement Grenoble France

**Keywords:** Antarctica, surface mass balance, precipitation, atmospheric rivers, detection, ice sheet mass balance

## Abstract

Atmospheric rivers (ARs) are efficient mechanisms for transporting atmospheric moisture from low latitudes to the Antarctic Ice Sheet (AIS). While AR events occur infrequently, they can lead to extreme precipitation and surface melt events on the AIS. Here we estimate the contribution of ARs to total Antarctic precipitation, by combining precipitation from atmospheric reanalyses and a polar‐specific AR detection algorithm. We show that ARs contribute substantially to Antarctic precipitation, especially in East Antarctica at elevations below 3,000 m. ARs contribute substantially to year‐to‐year variability in Antarctic precipitation. Our results highlight that ARs are an important component for understanding present and future Antarctic mass balance trends and variability.

## Key Points

- Atmospheric rivers (ARs) contribute around 13% ± 3% of the total Antarctic Ice Sheet (AIS) precipitation each year
- The relative contribution of ARs to precipitation is most substantial in East Antarctica
- ARs explain a large fraction (35%) of interannual variability in AIS precipitation

## 1 Introduction

The Antarctic Ice Sheet (AIS) is losing mass at an accelerated pace, with a tripling of mass loss (200 Gt yr^−1^ or Gigatons (10^12^ kg) per year) in recent years (2012–2017) relative to the early 1990s (Rignot et al., 2019; Shepherd et al., 2018). On top of that multi‐decadal AIS mass loss signal, which is primarily driven by ocean warming and subsequent ice shelf thinning and areal loss, and grounding line retreat, AIS mass balance varies substantially from year to year (Rignot et al., 2019; Wouters et al., 2013). These mass balance variations are determined by atmospheric processes, particularly snowfall, which is the primary input term of the AIS mass balance (Lenaerts et al., 2019). While annual snowfall rates on AIS are generally low (<200 mm per year), a substantial portion of the annual snowfall is associated with highly episodic marine air intrusions (Maclennan & Lenaerts, 2021; Nicolas et al., 2011) or synoptic‐scale cyclones (Dalaiden et al., 2020; Turner et al., 2019). Some of these systems are associated with long, narrow plumes of strong horizontal water vapor transport, referred to as atmospheric rivers (ARs; Zhu & Newell, 1998; Francis et al., 2020). While ARs are well known to impact certain mid‐latitude regions, particularly the west coast of the American continents, recent work has highlighted their importance for ice sheet mass balance. For example, Mattingly et al. (2020) showed that ARs in summer enhance surface melt and rainfall over the Greenland Ice Sheet. ARs have been found to strengthen extratropical cyclones moving into Antarctica, creating extreme wind, wave and ocean slope conditions that can trigger ice shelf calving events (Francis et al., 2021). Wille et al. (2019) demonstrated that most surface melt events on the West AIS are explained by ARs, as they bring relatively warm air masses from lower latitudes, sometimes as far as the subtropics (Terpstra et al., 2021). As surface melt and rain on the AIS is generally limited to the ice shelves (Johnson et al., 2021; Trusel et al., 2013), and nearly all meltwater refreezes in the firn (Gilbert & Kittel, 2021), AR impacts on the AIS are currently dominated by their contributions to snowfall. Considering that ARs are likely to become more impactful in the future (Payne et al., 2020), and AIS precipitation is expected to increase (Dalaiden et al., 2020; Lenaerts et al., 2016), it is essential to better constrain ARs and their impact on contemporary AIS surface mass balance. In situ observations on the East Antarctic escarpment have shown that ARs can contribute up to 80% of the annual snowfall (Gorodetskaya et al., 2014). In West Antarctica, seasonal surface height increases, as measured by satellite laser altimetry, can be attributed to unusually strong AR activity (Adusumilli et al., 2021). Most recently, Wille et al. (2021) used an AR detection algorithm to confirm that, while ARs only occur a few times per year along the Antarctic coastline, they contribute significantly to AIS snowfall especially in East Antarctica. A question that emerges from this previous work, in the framework of ongoing AIS mass loss, is: how much mass, in the form of precipitation, do ARs contribute to the AIS every year? Here we aim to address that question, using a combination of a polar‐specific AR algorithm and reanalysis precipitation products. Section 2 discusses the data and methods used in this study. Section 3 presents the results, and Section 4 provides a discussion of our findings and conclusions.

## 2 Data and Methods

### 2.1 MERRA‐2

In this work, we use output of the atmospheric reanalysis product MERRA‐2 (Gelaro et al., 2017) from the National Aeronautics and Space Association (NASA) for the period 1980–2020. In particular, we use total (snowfall + rainfall) precipitation fields at 3‐hourly (for the AR precipitation, see below) and monthly time resolution (for the total). We included rainfall in our study, since we consider it to be an input term to present‐day Antarctic mass balance. As rainfall is rare (Vignon et al., 2021), and most of the rain water instantaneously refreezes in the cold firn, we assume it adds mass to the AIS, along with snowfall. Effects of rain on firn thermal and density structure are not accounted for in this study. MERRA‐2 is selected because the snow accumulation (i.e., precipitation—sublimation) field over Antarctica compares most favorably to ice core accumulation records of multiple state‐of‐the‐art reanalysis products (Medley & Thomas, 2019).

### 2.2 AR Detection Algorithm

To detect ARs, we use the detection algorithm described in Wille et al. (2021), which uses the meridional component of the integrated water vapor (IWV) integrated water vapor transport (vIVT) fields between 37.5°S and 85°S from the MERRA‐2 atmospheric reanalysis. Previous studies using this AR detection algorithm have found similar AR detections when the algorithm is forced by other reanalysis data sets like ERA‐5, JRA‐55 (Japanese 55‐year Reanalysis) and Climate Forecast System Reanalysis (CFSR) (Wille et al., 2019, 2021). ARs are delineated by anomalously high (>98th percentile) vIVT values, and redetermined every 3 hr. If a filament of anomalously high vIVT values extends continuously for at least 20° in the meridional direction, then it is identified as an AR. It is a participating algorithm in Atmospheric River Tracking Method Intercomparison Project (ARTMIP; (Rutz et al., 2019; Shields et al., 2018)) and differs from many ARDTs in that is regionally specific to Antarctica, not a generalized global algorithm, and detects ARs at a lower frequency compared to other global algorithms. Following Wille et al. (2021), we use vIVT instead of IWV for AR precipitation attribution, given that the meridional moisture transport better reflects the dynamical processes associated to ARs. Unlike previous versions, the algorithm now extends down to 85°S to better capture AR associated precipitation over the Antarctic interior. In this updated version, there are slightly more AR detections north of 80°S mostly over the Antarctic continent, leading to marginally higher AR precipitation amounts and relative contributions (Figure S1). Due to the expanded domain, the AIS integrated total precipitation increases with ∼220 Gt yr^−1^ in the updated algorithm, and the AR precipitation increases with ∼40 Gt yr^−1^, yielding an unchanged relative contribution of ARs to total AIS precipitation (13%, see below).

**Figure 1:**
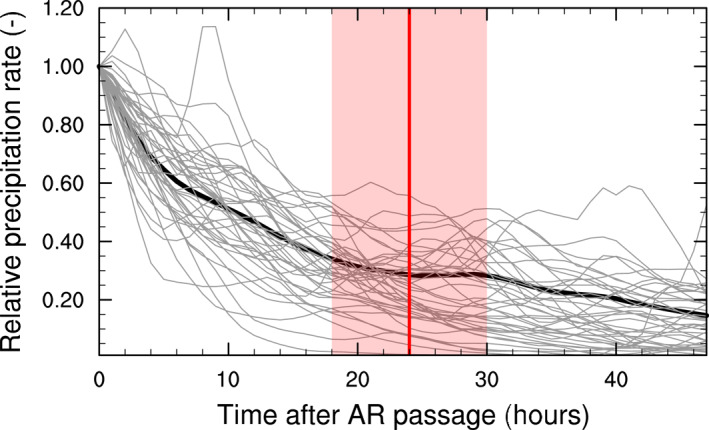
Mean precipitation rate as a function of time after atmospheric river (AR) passage, expressed relative to the last time step of AR passage, on the Antarctic Ice Sheet (AIS) for the year 2019. The thick black line denotes the mean of all ARs on the AIS, and the thin black lines show results for each individual glacier drainage basins (Shepherd et al., 2012). The vertical red line and shading show the 24 ± 6 hr time window that we have selected as the most suitable period to present our AR precipitation results. For clarity, this period can visually be interpreted using this figure: we include all times on the left side of the red line/shading to AR precipitation, and any precipitation on the right side of the red line/shading is excluded.

Next, we combine this AR detection catalog with MERRA‐2 total precipitation, and we define AR associated precipitation as precipitation that falls directly within each AR footprint, as well as in the 24‐hr (±6 hr) period after an AR has made passage. We selected this 24 ± 6 hr period after careful analysis of precipitation rates in MERRA‐2 during and after AR events. For this analysis, we used an hourly version of the AR detection algorithm (Collow et al., 2022), which provides more temporal detail but uses the same vIVT threshold method compared to the original 3‐hourly algorithm (see above). As shown by Collow et al. (2022), the hourly and three‐hourly AR algorithms produce very similar results in terms of integrated vapor transport globally, including over the AIS, so we expect similarly small differences in terms of AR detection. First, we examine precipitation rates in MERRA‐2 after the last time step when an AR was detected in the year 2019. We find that precipitation rates decrease by half in the first ∼10 hr after AR passage, after which their rate of decline slows (Figure 1). After 24 hr, on average more than 80% of cumulative precipitation has fallen since AR passage, and precipitation rates decrease very slowly afterward. Moreover, this choice of 24 hr has two practical advantages: (a) it is available in the 3‐hourly AR catalogs we use in this study and it is transferable to studies for which only daily precipitation rates are available; (b) this choice of time period is consistent with Wille et al. (2021). To quantify the uncertainty associated with this choice, we use the standard deviation of the time step when 80% cumulative precipitation is reached in each glacier drainage basin (Figure 1), which equals ∼6 hr.

Second, we checked the validity of this first method using a slightly different approach, and using our original 3‐hourly AR algorithm. We calculated the relative change in AIS‐integrated AR precipitation with each 3‐hourly increase in cut‐off time (e.g., 3 hr only accounts for precipitation until 3 hr after passage to the AR precipitation). While the relative increase initially increases steeply (e.g., 18% increase when using 6 hr past AR passage compared to using 3 hr past AR passage), the relative increase between each 3 hr increase quickly lowers, and is lower than 5% after 18 hr. This remaining small increase with a later cut‐off time is to be expected, and likely due to the residual AR‐related moisture being incorporated in mesoscale cyclogenesis over the AIS, the impact of a new AR as part of a series of ARs passing by the same location, and/or the general occurrence of AIS precipitation occurring outside the footprint of the detected ARs.

## 3 Results

**Figure 2:**
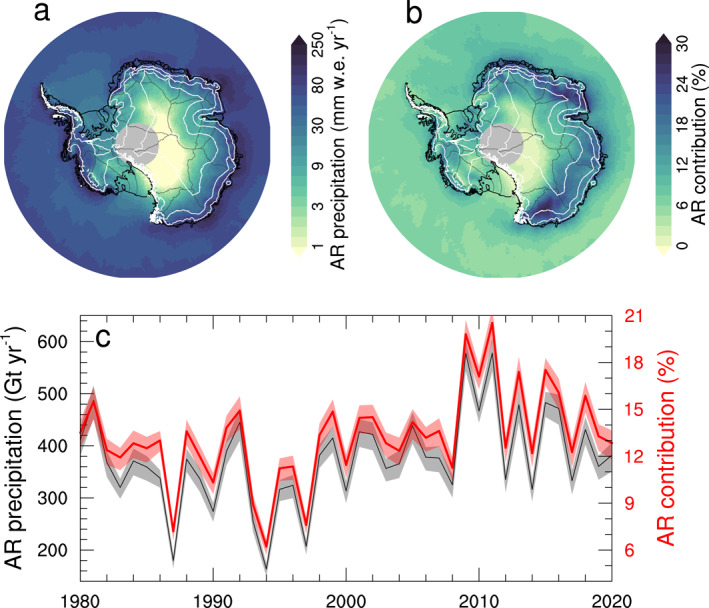
(a) 1980–2020 average precipitation attributed to atmospheric rivers (ARs) (mm w.e. per year); (b) 1980–2020 average relative contribution of AR precipitation (as shown in (a)) to the total annual precipitation; (c) Time series (1980–2020) of total Antarctic Ice Sheet (grounded ice sheet and ice shelves) AR precipitation (black, in Gt yr^−1^), and relative contribution of AR precipitation to total precipitation (in red, in %). The delineations of the drainage basins that are used further in this study are shown in thin black lines, and the grounding line and ice shelf boundaries are shown in thicker black lines.

First we focus our analysis on the AIS‐wide impact of ARs to precipitation. The annual precipitation that can be attributed to ARs varies from less than 1 mm w.e. per year on the Antarctic Plateau to >100 mm w.e. per year on the low‐elevation coastal zones (Figure 2a). While total annual precipitation exhibits a similar gradient, with high precipitation rates along the coast and very low precipitation rates in the interior (Lenaerts et al., 2019), the relative contribution of ARs to that total precipitation is generally highest at lower elevations of the grounded ice sheet. The highest contributions (>20%) are found in large parts of East Antarctica (Figure 2b), while relative contributions are lowest on the large Ross, Ronne‐Filchner, and Amery ice shelves, and in the high‐elevation (>3,000 m a.s.l.) interior. Remarkably, the relative contribution of ARs is markedly lower in the entirety of West Antarctica (generally <10%) compared to East Antarctica. Integrated over the full ice sheet (including ice shelves, and excluding areas poleward of 85°S), the AR precipitation equals 374 ± 90 Gt yr^−1^, equivalent to a 13% ± 3% of total precipitation (which equals 2,818 ± 138 Gt%yr^−1^). The relative importance of AR precipitation is slightly higher on the grounded ice sheet (14%) than on ice shelves (11%), and higher on East Antarctica (16%) than on West Antarctica (9%) and the Antarctic Peninsula (10%). These results imply that the relative impact of ARs on precipitation is at least an order of magnitude higher than their frequency on the AIS, which does not exceed 1%–1.5% (Wille et al., 2021).

Interannual variations in AR precipitation on the AIS are substantial, which is illustrated by the high (25%) ratio between the 1980–2020 AR precipitation standard deviation and mean, in comparison to the 5% ratio for total precipitation. This is further confirmed by the strong correlation (squared correlation coefficient *R* ^2^ = 0.96) between interannual variations of ice sheet integrated AR precipitation and its relative contribution to total AIS precipitation (Figure 2c). Detrended AIS AR precipitation and total precipitation are moderately correlated (linear slope = 0.98; *R* ^2^ = 0.35), indicating that ARs can explain more than a third of the interannual variability in total AIS precipitation, almost three times as much as ARs contribute to mean precipitation. Additionally, we find that the relative contribution of ARs to total AIS precipitation displays a small but discernible seasonal cycle (not shown), with a peak in winter and spring (>13.5%) and a relative minimum (<12.5%) in summer (December–February). Considering the substantial interannual variations, this seasonal cycle is nonetheless non‐significant.

On top of these interannual variations, MERRA‐2 suggests that total AIS precipitation has decreased during 1980–2020, albeit at a non‐significant rate (−1.5 ± 1.8 Gt yr^−2^ *p* = 0.4). AR precipitation, on the other hand, shows a clear and statistically significant upward trend (2.4 ± 1.1 Gt yr^−2^, *p* = 0.04). The opposite long‐term trends imply that the relative contribution of ARs to AIS precipitation has increased toward the later years of the time period (Figure 2), and relative AR contribution has increased substantially (0.1 ± 0.04% yr^−1^, *p* = 0.01), both for the grounded ice sheet and ice shelves. In 2009 and 2011, when multiple ARs hit the Dronning Maud Land area (Gorodetskaya et al., 2014), the AR contribution exceeded 20%.

**Figure 3:**
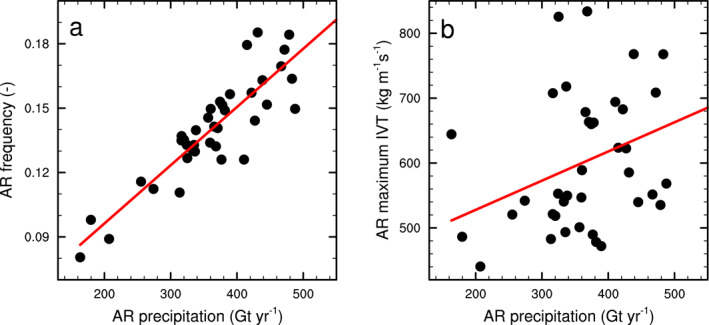
Annual Antarctic Ice Sheet (AIS) atmospheric river (AR) precipitation (1980–2019, horizontal axis) versus (a) annual AR frequency (1980–2019) on the AIS, defined here as the relative time an AR exists anywhere on the AIS; and (b) Annual mean (1980–2019) AR strength, approximated here by the mean IVT maximum found in all Antarctic ARs during each year.

An additional question emerging from our results is: what drives these variations in AR precipitation on the AIS? Both interannual variability and long‐term trend in AR precipitation are strongly (*R* ^2^ = 0.85, *p* < 0.01) correlated with the frequency of ARs on Antarctica (Figure 3a), indicating that years with more (less) ARs making landfall on AIS are also associated with more (less) AR precipitation. However, we also find a small but significantly positive (*R* ^2^ = 0.13, *p* = 0.02) correlation between AR precipitation and the annual average maximum IVT value in ARs on the AIS (Figure 3b), suggesting a potential link between the strength of ARs (as measured by IVT) on Antarctica and their precipitation.

**Figure 4:**
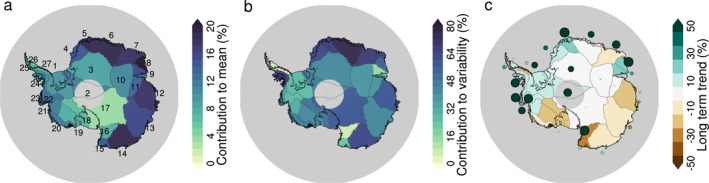
Relative contribution of atmospheric river (AR) precipitation to (a) the 1980–2019 average total precipitation; (b) the 1980–2020 interannual variability, defined as the percentage of explained variability of the best (positive) linear correlation between detrended annual AR and total precipitation; (c) the 1980–2019 relative change in total precipitation, with the dots showing relative change in AR precipitation (with same color scheme as for total precipitation, and size proportional to relative change). Note that basins that partly cover >85°S only include those areas <85°S in this analysis.

To further interpret the regional variability in the impact of ARs on Antarctic precipitation, we direct our analysis to individual glacier drainage basins. Figure 4a confirms that ARs contribute most to the total precipitation in the coastal East Antarctic basins (basins 5–9 and 12–15), with contributions of 12% up to 20% (basin 8). The coastal West Antarctic Ice Sheet (WAIS) and interior East Antarctic Ice Sheet (EAIS) basins show contributions of 10% and lower. The lowest AR contributions to precipitation are found on Ross shelf (<5%), where AR frequency is also lowest, but where summer ARs have been shown to induce surface melt (Wille et al., 2019). The spatial pattern in the extent to which ARs explain interannual variability (Figure 4b) largely reflects that of the AR contribution to the total, but the percentages are 3–4 times higher in most basins. In the coastal EAIS basins, 60%–75% of the variability in total precipitation is explained by ARs. Averaged across the EAIS, the explained variance is 66%, substantially higher than the WAIS (55%) and the Antarctic Peninsula (34%).

While we found an overall positive trend in AR precipitation over the AIS, along with a small negative trend in total precipitation, these long term trends vary considerably from basin to basin (Figure 2c). In terms of total precipitation, we find strongly negative trends in Wilkes Land and western WAIS (basins 12–20), and overall positive or negligible trends in other regions. AR precipitation trends are positive in most basins, and only negative in the western Wilkes Land region (basins 12 and 13). We find qualitative agreement between the trend signs in most basins, suggesting that the long‐term trends in AR precipitation partially explain the trends in total precipitation.

**Figure 5:**
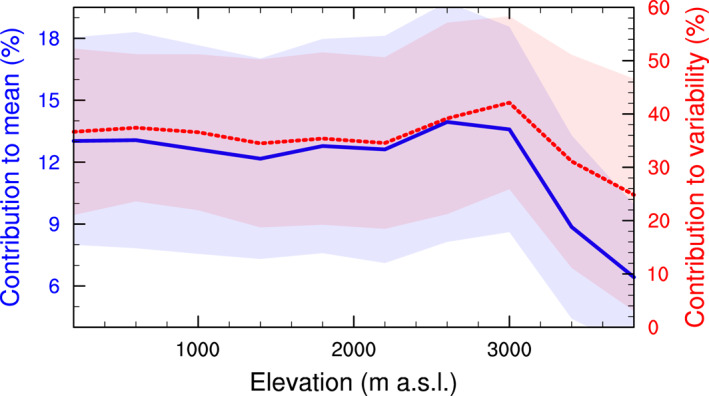
Relative contribution of atmospheric river (AR) precipitation to total precipitation (solid blue; left axis), and total precipitation variability (dashed red; right axis), as a function of elevation. The thick line shows the average of all basins, and the band indicates twice the standard deviation across all basins.

Finally, it is known that ARs, in part because of their remote, relatively warm source region and strong energetics, are able to penetrate deep in the interior of the ice sheet. We can validate this hypothesis using our results, as summarized in Figure 5. While the absolute precipitation rates associated with ARs clearly drop with elevation and distance to the coast (Figure 2a), their relative contribution to total precipitation remains remarkably constant from the coast all the way to 3,000 m a.s.l.. Above that elevation, the AR contribution drops sharply, but the associated uncertainties are large given that we have no data poleward of 85°S. Similarly, the contribution to total precipitation variability remains constant from 0 to 3,000 m a.s.l., and only slightly decreases above that elevation. This signal is fairly consistent among different basins, despite inter‐basin differences in values. This indicates that ARs are equally relevant to explaining annual accumulation and its interannual variability in many interior, dry areas below 3,000 m a.s.l. (where many ice cores are taken) as in low‐elevation, maritime locations.

## 4 Conclusions and Discussion

Our study combines a polar‐specific AR detection algorithm with MERRA‐2 precipitation rates to quantify the contribution of ARs to Antarctic snowfall. We find that, integrated over the ice sheet, ARs contribute around 13% to Antarctic snowfall. The contribution varies substantially from year to year and on a regional (glacier basin) scale, but is relatively constant throughout the seasons and across elevation. Our results provide a 41‐year long, spatially continuous record of AR precipitation on Antarctica. Given the substantial interannual variability of AR frequency and their precipitation impacts, our results aid to provide a broader context to short‐term, limited‐area contribution of ARs to annual precipitation on Antarctica (Adusumilli et al., 2021; Gorodetskaya et al., 2014; Terpstra et al., 2021).

We acknowledge that substantial uncertainties are associated with our findings, particularly as a result of our choice of AR detection algorithm and precipitation data set. On the other hand, we have several reasons to believe that our estimates are likely conservative. First of all, our precipitation product MERRA‐2 is a global, gridded atmospheric reanalysis that does not assimilate but parameterizes precipitation rates and has a modest horizontal resolution (0.5 × 0.625°). This likely leads to an underestimation of the highest precipitation rates, such as those associated with ARs, and thus an undercatch of the total precipitation assigned to an AR. Secondly, our assumed spatiotemporal footprint of an AR on precipitation likely underestimates its real footprint. We only assign precipitation directly underneath an AR to that system, while in reality, the precipitation field of an AR might be more expansive than that of the AR itself. The 24‐hr time window after AR passage we use (and justify) might miss precipitation that persists for more than 24 hr after the passage of strong AR systems (Wille et al., 2021). Thirdly, the AR detection algorithm used here is designed to capture high impact events, and likely misses weaker AR events more likely to be captured in global AR detection algorithms with lower thresholds. To further constrain ARs and their impact on Antarctica, future work should focus on using higher‐resolution precipitation products (e.g., ERA5 reanalysis, regional climate models), in conjunction with varied AR detection algorithms applied to that same product to estimate uncertainty based on the detection method. In addition, the AR detection algorithm could be refined, for example, by categorizing AR strength based on AR structure, size, vIVT threshold, and/or other conditions.

Current AR impacts in most regions of Antarctica are focused on snowfall, and thus ARs contribute positively to Antarctic surface mass balance. However, larger contributions of ARs to snowfall in East Antarctica compared to West Antarctica suggest that current AR impacts vary regionally. One hypothesis for this discrepancy is that West Antarctic ARs are less persistent, less meridionally expansive, and/or more of the “windy and warm” rather than “wet” type (Gonzales et al., 2020). Additionally, the zonal circulation around West Antarctica is better developed, and the position and strength of the Amundsen Sea Low, which is the dominant control of atmospheric circulation around West Antarctica (Turner et al., 2013), is extremely dynamic, both of which would limit the likelihood of the persistent atmospheric blocking needed for AR transport (Pohl et al., 2021). Further study is warranted to determine the AR flavors in different regions in Antarctica, and what impacts are associated to these different flavors. As global warming is likely to continue unabatedly in the future, leading to atmospheric temperature rise over Antarctica, AR strength and/or frequency might not only increase (Payne et al., 2020), but also their impact might shift. Particularly in the summer season and over coastal regions, ARs will bring enhanced potential of rainfall and surface melt at the expense of snowfall, which affects firn properties (Kuipers Munneke et al., 2014) and exacerbates the risk for ice slab formation, meltwater ponding, runoff, and ice shelf hydrofracture (Gilbert & Kittel, 2021). On the other hand, at higher elevations of the grounded ice sheet, ARs might bring even more enhanced snowfall in the future, aiding in mitigating ocean‐driven Antarctic mass loss.

## Supporting information

Figure S1

## Data Availability

MERRA‐2 precipitation data are available through the Goddard Earth Sciences Data and Information Services Center (hourly data at https://disc.gsfc.nasa.gov/datasets/M2T1NXLFO_5.12.4/summary, monthly means at https://disc.gsfc.nasa.gov/datasets/M2TMNXLFO_5.12.4/summary). The ARTMIP catalogues are available on the NCAR CGD gateway via https://doi.org/10.5065/D6R78D1M.
