# Supplementary material for: Contribution of Atmospheric Rivers to Antarctic Precipitation
Source: Geophys Res Lett. 2022 Sep 14;49(18):e2022GL100585. doi: 10.1029/2022GL100585 (PMC9539845; doi:10.1029/2022GL100585)
Supplement: Supplementary file 1 — Figure S1 [file GRL-49-e2022GL100585-s001.pdf]

GEOPHYSICAL RESEARCH LETTERS

Supporting Information for "Contribution of Atmospheric Rivers to Antarctic Precipitation"

Michelle L. Maclennan[1,*], Jan T. M. Lenaerts[1,*], Christine Shields[2],

Jonathan D. Wille[3]

[1]Department of Atmospheric and Oceanic Sciences, University of Colorado Boulder, Boulder CO, USA

[2]National Center for Atmospheric Research, Boulder CO, USA

[3]Institut des Géosciences de l'Environnement, Grenoble, France

* These authors have contributed equally to the work

Contents of this file

1. Figure S1

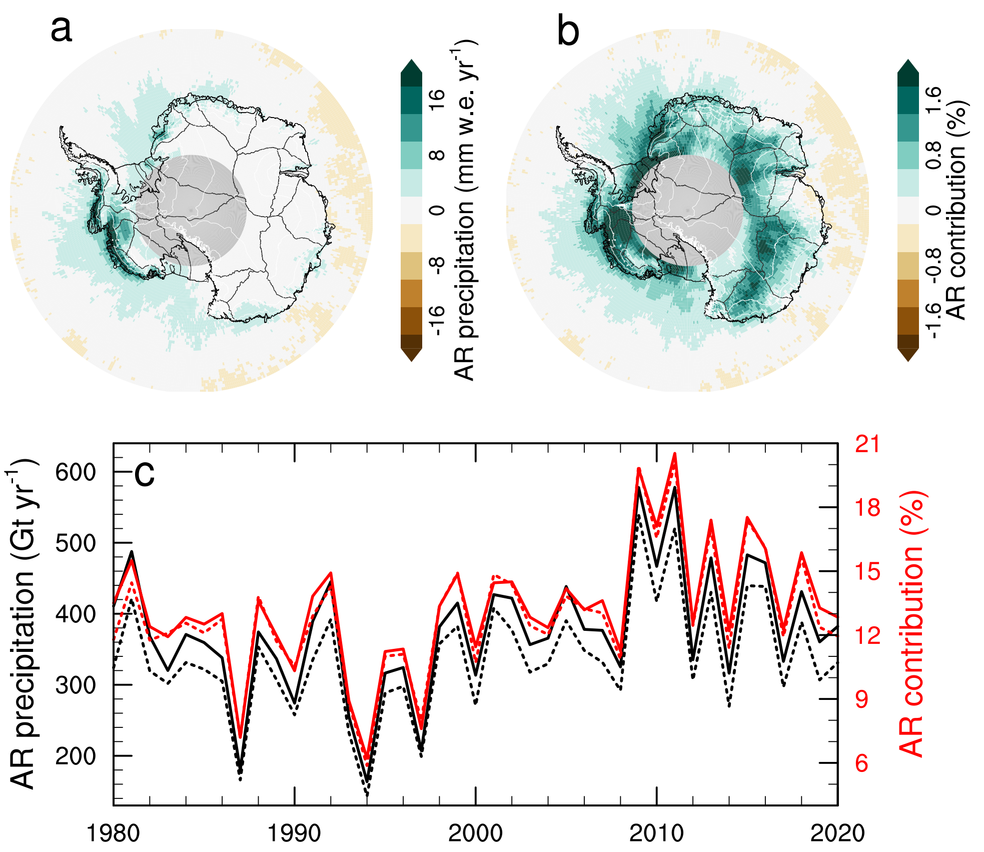

Figure S1. **a.** Difference of new algorithm (with a 85S boundary) and 'previous' (with a 80S boundary) algorithm in (a) annual AR precipitation; (b) annual relative contribution of AR precipitation to total precipitation. (c) Time series (1980-2020) of absolute AR precipitation (black) and relative contribution (red) in 'new' (solid) and 'previous' (dashed) algorithm.
